# Polyphenols and Sesquiterpene Lactones from Artichoke Heads: Modulation of Starch Digestion, Gut Bioaccessibility, and Bioavailability following In Vitro Digestion and Large Intestine Fermentation

**DOI:** 10.3390/antiox9040306

**Published:** 2020-04-10

**Authors:** Gabriele Rocchetti, Gianluca Giuberti, Franco Lucchini, Luigi Lucini

**Affiliations:** 1 Department for Sustainable Food Process, Università Cattolica del Sacro Cuore, 29122 Piacenza, Italy; gabriele.rocchetti@unicatt.it (G.R.); gianluca.giuberti@unicatt.it (G.G.); franco.lucchini@unicatt.it (F.L.); 2 Research Centre for Nutrigenomics and Proteomics (PRONUTRIGEN), Università Cattolica del Sacro Cuore, 29122 Piacenza, Italy

**Keywords:** polyphenols, foodomics, metabolomics, Caco-2, simulated digestion

## Abstract

Artichoke is a relevant source of health-promoting compounds such as polyphenols and sesquiterpene lactones. In this study, the bioaccessibility and gut bioavailability of artichoke constituents were evaluated by combining in vitro digestion and large intestine fermentation, metabolomics, and Caco-2 human intestinal cells model. Moreover, the ability of artichoke polyphenols to modulate the in vitro starch digestibility was also explored. An untargeted metabolomic approach based on liquid chromatography quadrupole-time-of-flight (UHPLC/QTOF) mass spectrometry coupled with multivariate statistics was used to comprehensively screen the phytochemical composition of raw, digested, and fermented artichoke. Overall, a large abundance of phenolic acids and sesquiterpene lactones was detected, being 13.77 and 11.99 mg·g^−1^, respectively. After 20 h of in vitro large intestine fermentation, a decrease in polyphenols and sesquiterpene lactones content was observed. The most abundant compounds characterizing the raw material (i.e., chlorogenic acid and cynaropicrin equivalents) showed an average % bioaccessibility of 1.6%. The highest % bioaccessibility values were recorded for flavonoids such as anthocyanin and flavone equivalents (on average, 13.6%). However, the relatively high bioavailability values recorded for flavonols, phenolic acids, and sesquiterpene lactones (from 71.6% up to 82.4%) demonstrated that these compounds are able to be transported through the Caco-2 monolayer. The phenolic compounds having the highest permeation rates through the Caco-2 model included low molecular weight phenolics such as tyrosol and 4-ethylcatechol; the isoflavonoids 3′-*O*-methylviolanone, equol 4′-*O*-glucuronide, and hydroxyisoflavone; together with the methyl and acetyl derivatives of glycosylated anthocyanins. Therefore, although human in vivo confirmatory trials are deemed possible, current findings provide insights into the mechanistic effects underlying artichoke polyphenols and sesquiterpenoids bioavailability following gastrointestinal and large intestine processes.

## 1. Introduction

Artichoke (*Cynara cardunculus* L. subsp. *scolymus* Hayek) is an important vegetable in the Mediterranean diet, being a good source of health-promoting constituents such as polyphenols, sesquiterpene lactones, soluble fibre (e.g., inulin), vitamins, and minerals [1]. According to literature, the most important and abundant (poly)-phenolic compounds in artichoke are isomers of caffeoylquinic acids, followed by flavones (such as glycosidic forms of luteolin and apigenin) and anthocyanins (mainly glycosidic forms of cyanidin) [2]. Sesquiterpene lactones (guaianolides) are another important class of bioactive compounds present in artichoke and are associated with several beneficial properties [3]. In this regard, cynaropicrin is the most abundant compound, showing a wide range of pharmacologic properties including antihyperlipidemic, antitrypanosomal, antimalarial, antifeedant, antispasmodic, anti-photoaging, and antitumoral action as well as activation of bitter sensory receptors and anti-inflammatory properties [4].

The actual content of phenolics and sesquiterpene lactones depends on cultivar, growth conditions, agronomic practices, and postharvest factors [2,5,6]. In addition, the beneficial effects of polyphenols are strongly related to their stability and availability during the digestion process [7] as well as to the interaction with the food matrix [8]. Previous information indicates a relatively low absorption of polyphenols, not exceeding plasma concentrations of 10 μM [9,10], likely related to the wide diversity in chemical structure among polyphenols able to affect their actual absorption in the gut [10]. Regarding artichoke, its bioactive constituents are reported to cross the gastric and intestinal barriers, reaching the human bloodstream [11]. However, undigested fractions from artichoke may deliver phenolic compounds to the colon, where they can undergo hydroxycinnamate metabolism by gut microbiota [12]. In particular, chlorogenic acid is hydrolyzed by colon enzymes to aromatic acid metabolites (such as coumaric or benzoic acids), while caffeic acid is converted to dihydrocaffeic and dihydroferulic acid [12]. Indeed, artichoke is reported to contain an indigestible fraction (mainly fibre) that is known to acts as a carrier of phenolic compounds [13,14].

Together with a(n) (in)direct antioxidant effect, polyphenols have also shown a good potential to modulate starch digestion, hence having an antidiabetic potential. In this regard, artichoke (as member of Asteraceae family) was found to possess anti-obesity properties [11] that have been connected to the potential inhibition of starch-digestive enzymes. Recently, Turkiewicz et al. [15] showed that the inhibition of digestive enzymes was strictly related to the cultivar or hybrid analysed. In addition, new cultivars and hybrids of artichoke have been also reported as effective inhibitors of neurodegenerative enzymes (such as cholinesterase), thus highlighting the pharmacological potential of extracts from artichoke.

Regardless from the activity considered, the aforementioned biological effects ascribed to either phenolics or sesquiterpene lactones are known to be dramatically affected by digestion and fermentation processes. The fraction actually reaching the large intestine plays a pivotal role in determining gut health by either directly providing antioxidant compounds or by positively modulate gut microbiota [16,17]. The bioaccessibility of phenolics and the potential antioxidant effects of artichoke along the gastrointestinal tract were previously investigated both in vivo and in vitro [18,19]. However, scientific literature is limited regarding sesquiterpene lactones and still lacks the comprehensive screening of phenolic metabolites arising from the large intestine processes and being potentially bioavailable. Therefore, this work aimed to investigate the modifications induced by in vitro static digestion followed by in vitro large intestine fermentation processes on both polyphenols and sesquiterpene lactones from artichoke, followed by the evaluation of their bioaccessibility/bioavailability into the gut and the potential modulation of the different starch fractions. Moreover, the potential of artichoke extracts (AE) to modulate the different starch fractions during in vitro digestion has been tested. Once gathered, the information targeted by the present work will help to better support the health-promoting properties of artichoke.

## 2. Materials and Methods

### 2.1. Samples and Extraction Procedure

Artichoke samples were purchased as fresh heads from a local market and stored at 4 °C until used. Samples (1.0 g) were extracted in 10 mL of 80% methanol using a homogenization system (Ultra-turrax Ika T25, Staufen, Germany; 20,000 rpm; 5 min) as previously reported [2]; the methanol was then evaporated by a vacuum rotary evaporator, and the solution was diluted to the initial volume with water. Thereafter, supernatants were filtered (0.22 µm cellulose syringe-filters) and stored at −18 °C.

### 2.2. In Vitro Modulation of Starch Fractions by Artichoke Extract

The inhibitory effect of AE towards the activity of starch hydrolyzing enzymes was assessed through the measurement of nutritionally important starch fractions [20]. In this context, to better simulate the effect in foods, previously obtained AE were mixed with maize starch (powder equivalent to 100 mg dry matter of starch; SKU code: S4126, Sigma-Aldrich, Milan, Italy) and cooked simultaneously (i.e., starch-to-water ratio of 1:2 *w/v*; 100 °C; 15 min) before adding the starch hydrolyzing enzyme mixture. Native maize starch was weighed into capped glass tubes, and increasing levels of AE were incubated as a percentage of the total starch (i.e., 0%, 25%, 50%, and 100%) [21].

The rapidly digestible (RDS), slowly digestible (SDS), and resistant starch (RS) fractions were measured by in vitro enzymatic incubation as previously detailed [20]. The procedure consisted of a gastric digestion with pepsin (SKU code: P7000; Sigma-Aldrich^®^ Co., Milan, Italy) at 37 °C for 30 min. To simulate the pancreatic digestion phase, the pH was increased to 5.2 with 0.1 M sodium acetate buffer and an enzyme mixture (i.e., 2.05 mL; amylase activity of about 7000 U·mL^−1^) composed of pancreatin (Merck 7130, Merck KGaA, Darmstadt, Germany), amyloglucosidase (SKU code: A7095, Sigma-Aldrich^®^ Co., Milan, Italy), and invertase (SKU code: I4504, Sigma-Aldrich^®^ Co., Milan, Italy) was used. Liquid aliquots were carefully taken after 20 and 120 min of pancreatic incubation, absolute ethanol was added to stop the enzymatic hydrolysis, and the amount of the released glucose was quantified after centrifugation with a glucose oxidase kit (GODPOD 4058, Giesse Diagnostic snc, Rome, Italy). Results were expressed as RDS and SDS (i.e., starch converted from glucose after 20 and 120 min of incubation). The RS content was measured as the difference between total starch and the starch hydrolyzed at 120 min of incubation. Analyses were conducted in triplicate, and results were expressed as g·100 g^−1^ dry starch.

### 2.3. In Vitro Gastrointestinal Digestion and Large Intestine Fermentation Process

Raw artichoke samples were subjected to an in vitro static human digestion procedure [22]. Simulated salivary fluid (SSF), simulated gastric fluid (SGF), and simulated intestinal fluid (SIF) were prepared following the scheme previously reported in literature [22]. Briefly, the oral phase involved the SSF (1:1 *w/v*) with α-amylase from human saliva (SKU code: A1031; Sigma-Aldrich; 75 U·mL^−1^) at 37 °C for 2 min. The oral bolus was then diluted with 10 mL of SGF at pH 3.0 containing pepsin (SKU code: P7012; Sigma-Aldrich; 2.000 U·mL^−1^) at 37 °C for 120 min. The gastric chime was then diluted with 20 mL of SIF at pH = 7.0 containing pancreatin (SKU code: P7545; Sigma-Aldrich; 100 U·mL^−1^ based on trypsin activity) and bile salts (SKU code: B8631; Sigma-Aldrich; 10 mM) for 120 min. During the in vitro digestion, appropriate amounts of HCl (1 M) and NaOH (1 M) were added for pH adjustment. The in vitro digestion was stopped by cooling on ice. Unhydrolyzed solid residues were collected after centrifugation at 10,000× *g* for 12 min, air-dried overnight, and ground to pass through a 60-mesh screen. Samples were incubated in triplicate in three separate runs.

The in vitro large intestine fermentation was conducted on the solid residues as reported by Rocchetti and coauthors [23]. Fresh faeces were collected from four growing pigs (35.4 ± 2.85 kg body weight; 3–4 months of age). Pigs had free access to water and were fed a commercial diet devoid of antibiotics. Freshly voided faecal samples were captured after physiological defecation, pressed in sterile airtight plastic syringes, and kept at 39 °C. Faecal samples were used within 20 min after collection. Unhydrolyzed artichoke residues were weighed (1 g) in triplicate into 125 mL amber glass bottle filled with 10 mL of a filtered buffer solution prepared according to Williams et al. [24]. The CO_2_-saturated fermentation medium contained 0.05 g mL^−1^ of fresh faeces obtained by pooling equal amounts (wet weight) of faeces from each animal. Three bottles without substrate were used as control of background fermentation. Sample manipulation and incubation were done under continuous CO_2_ flushing (technical grade: 5.5; from SAPIO, Monza, Italy). Bottles were sealed with a rubber stopper and placed in a shaking water bath (60 rpm) at 37 °C for 20 h [25]. Samples were then immersed in ice to stop the reactions and centrifuged at 10,000× *g* for 12 min to remove insoluble particulate. Supernatants were collected and stored at −4 °C for further analysis.

### 2.4. Cell Culture and In Vitro Bioavailability of Polyphenols and Sesquiterpene Lactones

Each supernatant (0.5 mL) was adjusted to pH 6.8 by adding the required volume of 300 mM NaOH (about 75 µL) and brought to a volume of 2 mL with Hanks’ balanced salt solution (HBSS pH 6.8, final dilution ratio 1:4). The diluted samples were finally sterilized by filtration through a 0.22 µm cellulose syringe filter and used in Caco-2 permeability assay. Caco-2 human colon carcinoma cells were obtained from the American Type Culture Collection and cultured in Dulbecco’s modified Eagle’s medium with 4.5 g/L glucose (DMEM-high) supplemented with 10% fetal calf serum (FCS), 5 mmol·L^−1^ L-glutamine, and 50 mg·mL^−1^ gentamycin. Cultures were maintained at 37 °C in a 5% CO_2_ atmosphere. The medium was changed every second day, and cells were split by trypsinization when reaching the confluence. Following the second passage in a flask, cells were trypsinized and seeded in 0.4 µm tissue culture inserts in 12-well plates (TC-inserts, Sarstedt). Each insert was filled with a suspension of 6 × 10^4^ cells in 0.5 mL of culture medium, whilst the corresponding well received 1.5 mL of the same medium without cells. The medium was changed every second day for 18 days. According to this protocol, a mature epithelial layer is obtained in 15 days [26]. In the present study, cell monolayers were employed on day 18th and the complete occlusion of the filter by a mature epithelial layer was validated ex post choosing selected compounds as internal reference (see the Results section). To perform permeability assays, the medium was removed from the insert and the recipient well and replaced by the same volumes of HBSS for a preliminary rinse. Thereafter, the solution in the outer well (basolateral side of the epithelium) was replaced by the same amount of fresh HBSS, while the solution in the insert (apical side) was replaced with 0.5 mL of the fraction to be tested. Cells were returned to 37 °C and incubated for 60 min. Following incubation, the solutions from the insert and the external well were recovered and stored at −20 °C until analysis.

### 2.5. Profiling of Polyphenols and Sesquiterpene Lactones by UHPLC-ESI-QTOF Mass Spectrometry

Thereafter, the AE and supernatants collected after 20 h of large intestine fermentation together with fractions from Caco-2 cellular absorption assays were screened by untargeted ultrahigh-pressure liquid chromatography coupled to a quadrupole-time-of-flight mass spectrometer (UHPLC-ESI/QTOF-MS). In particular, a 1290 series liquid chromatograph coupled to a G6550 iFunnel mass spectrometer through a Dual Electrospray Jet Stream ionization system (all from Agilent technologies, Santa Clara, CA, USA) was used. The mass spectrometer was set to operate in SCAN mode and positive polarity, acquiring ions in the range of 100–1200 *m/z* and in extended dynamic range mode. The analytical conditions for the analysis of bioactive compounds in these food matrices were optimized in previous experiments [27]. Briefly, the chromatographic separation was achieved using a Knauer Blue Orchid C18 column (100 × 2 mm i.d., 1.8 μm) and a mixture of water and acetonitrile (both LC-MS grade, VWR, Milan, Italy) as mobile phase. A gradient separation, from 6% acetonitrile to 94% acetonitrile within 33 min, and a flow rate 220 μL·min^−1^ were used for the chromatographic separation. The injection sequence was randomized, with three replicates (6 μL injection volume) for each sample. Raw data were processed using the Agilent Profinder B.06 software, according to the “find-by-formula” algorithm. Monoisotopic accurate mass and the entire isotope pattern (isotopic spacing and isotopic ratio) were used to achieve high confidence in annotation. A custom database was built and used as reference, combining phenolic compounds and their metabolites from Phenol-Explorer 3.6 (http://phenol-explorer.eu/) and the most important sesquiterpene lactones reported on FoodDB (foodb.ca) for artichoke. The annotation workflow used allowed compounds identification according to level 2 (i.e., putatively annotated compounds), with reference to the COSMOS (COordination of Standards in MetabolOmicS) initiative [28]. Annotated compounds were retained when passing post-acquisition filters (mass accuracy tolerance 5 ppm, retention time shift < 0.1 min, presence in 100% of replications within one treatment) and threshold (min abundance 10.000 units), with plausible chromatographic peak features and showing intensities significantly differing from the control (faeces only).

Thereafter, in order to provide semiquantitative information, phenolic compounds and sesquiterpene lactones were ascribed into classes and subclasses and then cumulative intensity for each subclass was converted to mg phenolic equivalents g^−1^ dry matter (DM) by means of calibration curves obtained from pure standard compounds (from Extrasynthese, Lyon, France). In this regard, the standards used were sesamin (lignans), chlorogenic acid (phenolic acids), cyanidin (anthocyanins), tyrosol (tyrosols and low-molecular-weight phenolics), resveratrol (stilbenes), catechin (flavan-3-ols), quercetin (flavonols), luteolin (flavones and isoflavonoids), and cynaropicrin (sesquiterpene lactones). Calibration curves were built using a linear fitting (unweighted and not forced to axis-origin) in the range 0.05–500 mg·L^−1^; a coefficient of determination R^2^ > 0.98 was used as acceptability threshold for calibration purposes.

The % of bioaccessibility and bioavailability of the different phytochemical classes were finally calculated as:

*% Bioaccessibility*: Total content per class (mg equivalents 100 g^−1^) of fermented samples/total content per class (mg equivalents 100 g^−1^) starting material;

*% Bioavailability*: Total content per class (mg equivalents 100 g^−1^) of permeated samples/total content (mg equivalents 100 g^−1^) of fermented samples.

### 2.6. Statistical Analysis

Normal distribution of the data was verified by the Shapiro–Wilk test before statistical analysis. The starch fraction content was then analyzed by adopting a completely randomized design using the general linear models (GLM) procedure of SAS 9.3 (SAS Inst. Inc., Cary, N.C., USA). The fixed effect of the model was the AE inclusion level (*n* = 4). The means were post hoc compared using Duncan’s test (*p* < 0.05). One-way analysis of variance (ANOVA; *p* < 0.05) was performed using PASW Statistics 26.0 (SPSS Inc.) to investigate significant differences (*p* < 0.05) in semiquantitative values of each representative class of compounds. Duncan’s post hoc test was then performed to identify homogenous subclasses.

Normalization of metabolomics-based data was done using the Agilent Mass Profiler Professional B.12.06 software, as previously reported [23,27]. Afterwards, an unsupervised hierarchical cluster analysis (HCA, Euclidean distance) was created in order to group samples according to intrinsic similarities in their measurements. Finally, analysis of variance (ANOVA; *p* < 0.01, Bonferroni multiple testing correction) was combined to fold-change analysis (threshold > 5, considering log2 normalized values) in order to produce Volcano Plots on each possible comparison.

## 3. Results and Discussion

### 3.1. Characterization of Artichoke Extract by UHPLC-QTOF Mass Spectrometry

In this work, a comprehensive UHPLC-QTOF mass spectrometry approach was used to comprehensively investigate the phytochemical profile of artichoke head, focusing on polyphenols and sesquiterpene lactones. Overall, 365 compounds were putatively annotated in our experimental conditions, with the highest frequency for flavonoids (144 compounds) compared to the other classes. Moreover, phenolic acids accounted for 103 compounds, followed by tyrosol equivalents, lignans, and stilbenes (being 62, 28, and 23 annotations, respectively). Interestingly, among the five sesquiterpene lactones annotated, we found a large abundance of dehydrocynaropicrin, followed by grosheimin and cynaratriol. A detailed list of all polyphenols and sesquiterpene lactones annotated in artichoke raw extracts is provided as Table S1, together with the corresponding composite mass spectra.

From a quantitative point of view, 23 compounds were the most abundant in their chemical class (Table S1). In this regard, among phenolic acids, isomeric forms of caffeoylquinic and caffeic acids were the most representative hydroxycinnamics while isomeric forms of hydroxybenzoic acid (i.e., 2, 3, and 4) characterized the hydroxybenzoics. In addition, isomeric forms of malvidin were the most abundant anthocyanins while tetramethylscutellarein characterized the flavones group. Interestingly, we observed other abundant compounds, such as sesamol (belonging to lignans) and tetramethoxystilbene derivatives (stilbenes). Overall, the untargeted approach used allowed to explore in a deeper way the polyphenol composition of artichoke heads. In fact, according to literature, the most important compounds characterizing this matrix are isomeric forms of caffeoylquinic acid, chlorogenic acid, cynarin, isomers of dicaffeoylquinic acid, apigenin 7-*O*-glucuronide, luteolin, luteolin 7-*O*-glucuronide, and cyanidin caffeoylglucosides [19,29]. Despite being aware that a number of pre- and postharvest factors determine the actual phenolic and sesquiterpene lactone compounds in artichoke, untargeted metabolomics allowed us to deeply investigate the phytochemical composition of artichoke extracts. In a previous work, Rouphael et al. [2] described the phenolic compounds and sesquiterpene lactone profiles in leaves of different artichoke cultivars. Using UHPLC-QTOF mass spectrometry, the authors showed quite diverse phenolic profiles, including flavonoids, hydroxycinnamic acids, tyrosols, and lignans, with grosheimin characterized by the highest relative abundance values [2]. However, it is important to remember that polyphenol and sesquiterpene distribution in plants is strictly dependent on environmental conditions, crop management strategies, biotic stressors, postharvest handling storage, industrial and domestic processing, harvest time, cultivar type, and plant tissue analyzed [5,30]. These factors were not considered in this work because the main aim was to highlight the potential of artichoke as “food-system” focusing on the ability to deliver bioaccessible phytochemicals to the gut, together with the evaluation of possible inhibitory activity on digestive enzymes.

**Figure 1:**
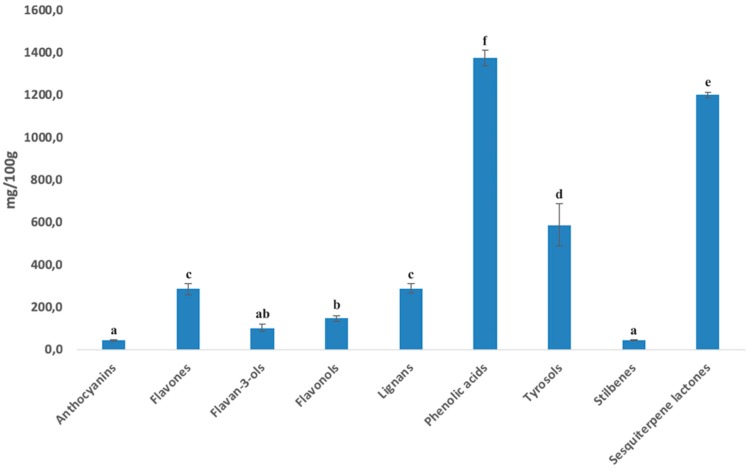
Semiquantitative cumulate values (expressed as mg·100g^−1^ DM) for the different phenolic classes and for total sesquiterpene lactones, as resulted from UHPLC-quadrupole-time-of-flight (QTOF)-MS profiling.

Afterwards, by using the curves from pure standard compounds, semiquantitative values were calculated for each class and the cumulative contents are provided in Figure 1 as mg equivalents 100 g^−1^ DM.

As can be observed from the figure, phenolic acids and sesquiterpene lactones were the most abundant compounds (*p* < 0.05), being 1376.90 and 1199.87 mg·100 g^−1^, respectively, followed by tyrosol equivalents (587.83 mg·100 g^−1^) and lignans (288.30 mg·100g^−1^). Flavones were the most abundant (*p* < 0.05) among flavonoids (285.82 mg·100 g^−1^), while anthocyanins and stilbenes accounted for the lowest concentrations (on average 45.42 mg·100 g^−1^; *p* < 0.05). Starting from such a diverse composition of artichoke head extracts, the following investigations aimed to monitor the impact of AE on starch fractions and the fate of both polyphenols and sesquiterpene lactones in the distal tract of the gut by simulated in vitro large intestine fermentation.

### 3.2. Modulation of Different Starch Fractions by Artichoke Extract

The modulation of the starch fraction by increasing level of AE is provided in Table 1.

On average, cooked maize starch used as control (i.e., 0% AE) showed the highest RDS (84.4 g·100 g^−1^ dry starch) and the lowest RS (1.1 g·100 g^−1^ dry starch) contents (*p* < 0.05). Similar results have been reported by Camelo-Méndez et al. [31]. During heating in the presence of water, starch undergoes a series of phase transitions (i.e., gelatinization process) which can disrupt the inherent structures of native starch granules, thereby increasing the susceptibility of starch to the in vitro amylolysis [32,33]. When compared to the control, the addition of AE reduced (*p* < 0.05) the RDS content in a dose-dependent manner, with average reduction percentages of about −2.2%, −3.8%, and −8.3% at AE addition levels of 25%, 50%, and 100% of the total starch, respectively. In addition, at the highest AE inclusion level (i.e., 100% of the total starch), the SDS increased by about 47.9% (*p* < 0.05), whereas the RS fraction increased by about 75.2% (*p* < 0.05) compared to control.

Several indications suggested that RDS fraction can induce a fast increase in blood glucose and insulin levels in humans whereas the SDS can result in a prolonged release of glucose into the blood stream [34]. In addition, a number of physiological benefits have been ascribed to RS, including but not limited to a positive impact on blood glucose and lipid profiles along with a possible role on bowel health maintenance [35]. The modulation of the starch fractions following the addition of AE can be related to the inhibitory effect of polyphenols on starch-digestive enzymes [36]. In line with present findings, several indications reported that the addition of phenolic extract from different sources can modulate the in vitro digestibility both in raw and gelatinized starches [37]. Accordingly, previous in vitro evidences demonstrated that polyphenol-rich extracts resulting from the digestion of breads enriched with increasing amounts of artichoke stem powder exerted an inhibition activity towards glycolytic enzymes as a function of the level of addition [38]. Such effect may be induced by two mechanisms, being the modulation of the in vitro starch digestibility via interactions between polyphenols and starch-digestive enzymes and/or through the formation of inclusion and noninclusion complex with starch on cooking with limited enzyme accessibility [39]. In this context, part of the phenolic compounds characterizing artichoke extract, mainly caffeoylquinic acid and dicaffeoylquinic acid derivatives, have been reported to modulate the activity of α-glucosidase enzymes [40].

### 3.3. In Vitro Bioaccessibility and Bioavailability of Polyphenols and Sesquiterpene Lactones

**Figure 2:**
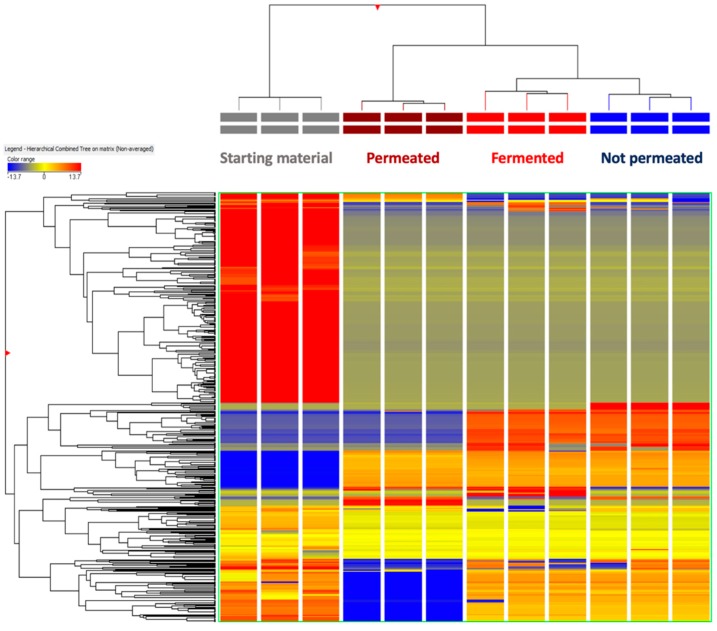
Unsupervised hierarchical cluster analysis of phenolic and sesquiterpene lactone profiles gained from starting (undigested) material, fermented samples (at 20 h of in vitro large intestine fermentation) as well as permeated and not permeated fractions from Caco-2 cellular trial: The clusters were produced from the fold-change based heatmap, assuming a Euclidean distance.

The fate and bioavailability of polyphenols and sesquiterpene lactones from artichoke heads was investigated after in vitro gastrointestinal and large intestine fermentation by coupling untargeted metabolomics and a Caco-2 cellular-based model. In particular, as can be observed from unsupervised hierarchical cluster analysis (HCA (Figure 2)), a clear modification of the phytochemical profiles was highlighted comparing raw to in vitro digested and fermented samples. In fact, two main clusters characterized the HCA heat map (Figure 2); the first cluster consisted of sample replicates of the raw material, whilst the second cluster was composed by aliquots collected at the end of the in vitro digestion and large intestine fermentation process together with permeated and not permeated fractions from Caco-2 cellular-based model.

Hierarchically, the permeated fraction could be then clustered apart in this second cluster, suggesting that bioavailability differed from class to class of compounds. The unsupervised HCA was effective in naively depicting the main changes of both polyphenols and sesquiterpene lactones following the simulated in vitro digestion and fermentation processes. In fact, it was clear that some parent compounds characterizing the raw material tended to disappear during in vitro large intestine fermentation, thus allowing new metabolites to be formed, likely due to the microbiota processing and enzymatic reactions. From a qualitative point of view, we found that in vitro large intestine fermentation determined a general reduction in the compounds annotated (234 compounds), recording 110 flavonoids, 21 lignans, 39 tyrosol equivalents, 43 phenolic acids, 4 sesquiterpene lactones, and 16 stilbenes. The compounds annotated during the in vitro large intestine fermentation are reported in Table S1, together with their corresponding composite mass spectrum. Overall, at 20 h of in vitro large intestine fermentation, we observed the formation of the enterolignans enterolactone and enterodiol (Table S1). These compounds are formed from plant lignans by microorganisms in the human colon [41]. Therefore, their detection is consistent with the presence of plant lignans in the starting material; in fact, according to literature [42], the final microbial products of syringaresinol and pinoresinol, with several intermediates (such as secoisolariciresinol), are enterodiol and its oxidized form, namely enterolactone. Secoisolariciresinol can also be converted to matairesinol, from which only enterolactone is obtained from microbial catabolism [42]. Regarding chlorogenic acids in the raw material, we found several metabolites related to their processing by gut microbiota, such as caffeic acid and isomeric forms of hydroxyphenylpropionic acid (Table S1). Overall, untargeted metabolomics allowed to observe also several metabolites related to flavonoids catabolism; in particular, the most represented catabolic pathways were those of flavones and isoflavonoids, recording several glucuronides likely arising from microbial catabolic activity.

There is still a lack of information regarding the modifications of polyphenols during simulated gastrointestinal processes and especially on the factors potentially influencing their bioaccessibility/bioavailability. Overall, bioaccessibility is defined as the amount of nutrients or phytochemicals released from the food matrix in the lumen of the gastrointestinal tract, where they become available for absorption into the body [8,23]. Although presenting physiological limitations, in vitro digestion and fermentation methods are really useful to better understand the fate of phytochemicals in the whole gastrointestinal tract together with their potential bioaccessibility. The untargeted metabolomics profiling allowed confirmation that these compounds become bioaccessible in the large intestine, although with low % bioaccessibility values detected (Table 2).

Overall, the higher % bioaccessibility values were recorded for anthocyanins (14.2%) and flavones (13%), followed by lignans (7.7%). The lowest bioaccessibility values were recorded for the two most characteristic classes of bioactives in artichoke, namely sesquiterpene lactones (1.6%) and phenolic acids (1.6%). Looking at the information available in literature, D’Antuono et al. [19] showed that in vitro digestive recoveries (bioaccessibility) of total polyphenols from artichoke heads reached 56%, with the highest bioaccessibility recorded for chlorogenic acid (70%), followed by isomers of dicaffeoylquinic acids (on average 45%). Therefore, these compounds have been described as potentially sensitive to gastric and small intestinal digestive conditions. According to what was observed for phenolic acids, we found a strong impact of in vitro gastrointestinal process on sesquiterpene lactones content (Table 2); in fact, cynaropicrin equivalents decreased from 1199.87 to 19.94 mg/100g (bioaccessibility = 1.6%). Recently, Colantuono et al. [38] evaluated the potential bioaccessibility and functionality of polyphenols and cynaropicrin from breads enriched with artichoke stem; the authors showed that 82% of polyphenols and that 74% of cynaropicrin were bioaccessible in duodenal step whilst 88% of caffeic acid was solubilized during the colon step. As reported in literature, some sesquiterpenoids are sensitive to a wide range of chemical and biological modifications; therefore, according to Colantuono et al. [38], it is plausible to postulate their degradation by the simulated physiological conditions rather than a not effective extraction by the aqueous digestive fluids [43]. Further ad hoc studies focusing on sesquiterpenoids are needed to clarify the mechanisms underlying sesquiterpene lactones bioaccessibility. This would be of particular interest for the function foods area, considering that cynaropicrin and other related compounds possess a wide range of pharmacological properties that may work from the intestine, also through activation of bitter taste receptors [38]. Overall, our findings are difficult to compare with existing literature, considering the differences existing in both analytical approaches and in vitro large intestine fermentation conditions. Moreover, from our data, it is not possible to discern between the food matrix-carrier contribution (i.e., impact of dietary fibre) and microbial transformations of phenolics in providing the bioaccessibility values observed. In fact, it could be a combination of both factors, although a clear contribution of microbial processes can be postulated by looking at specific phenolic metabolites detected (Table S1).

Thereafter, the bioavailability of both polyphenols and sesquiterpene lactones from artichoke was evaluated using Caco-2 cells monolayers as a model of absorption in the large intestine. The basolateral side (permeated fraction) that simulated the blood plasma compartment was analyzed by using UHPLC-QTOF mass spectrometry with the objective to gain insight into polyphenols and sesquiterpenoids uptake and bioavailability. The results obtained are presented in Table S1. As can be observed from Table S1 (Table S1), 129 compounds were detected into the basolateral (permeated) fraction, with the highest % bioavailability values recorded for cynaropicrin equivalents (sesquiterpene lactone, 82.4%), followed by catechin and chlorogenic acids equivalents (flavan-3-ols and phenolic acids, being 71.6% and 67.6% respectively). Notably, both sesquiterpene lactones and flavan-3-ols were the classes recording the lowest bioaccessibility values. Finally, volcano plot analysis (combining ANOVA and fold-change analyses) was employed to select those compounds significantly changing between permeated and fermented fractions. Overall, 69 compounds (including some isomeric structures; Table S1) were detected, with a clear down-accumulation trend in the permeated compared to the fermented fraction (−84%, on average), thus confirming the outcomes obtained from unsupervised HCA (Figure 2). Looking at the phenolics having the highest permeation rates, low molecular weight phenolics such as tyrosol and 4-ethylcatechol; the isoflavonoids 3′-*O*-methylviolanone, equol 4′-*O*-glucuronide, and hydroxyisoflavone; together with the methyl and acetyl derivatives of glycosylated anthocyanins were the most represented compounds among the Caco-2 bioavailable fraction (Table S1).

## 4. Conclusions

Artichoke has been confirmed to be a nutritionally relevant source of both phenolic compounds and sesquiterpene lactones. Our findings, based on in vitro digestion and fermentation and on metabolomics, highlighted that only a limited portion of the initial amounts become bioaccessible in the large intestine. Nonetheless, phenolics underwent an extensive transformation during the digestion and fermentation processes, resulting in the appearance of lower molecular weight phenolic compounds. Despite the low bioaccessibility values recorded, Caco-2 bioavailability assays allowed recording good bioavailability values, in particular for sesquiterpene lactones and lower molecular weight phenolics. These results highlight the importance of considering also the pivotal role played by digestion and fermentation process in addition to the content in raw commodities. Despite in vivo confirmations being always recommended, the proposed approach may provide useful insight into the contribution of digestion-related processes.

## Figures and Tables

**Table 1:** Starch fractions content of cooked normal maize starch (g 100 g^−1^ dry starch) in the presence of increasing level of artichoke water extract (AE).

	AE Addition Level ^1^ (% of Addition)			
	0%	25%	50%	100%
Rapidly digestible starch (RDS)	84.39 ± 0.76 ^c^	82.61 ± 1.94 ^b^	81.19 ± 1.42 ^b^	77.38 ± 1.61 ^a^
Slowly digestible starch (SDS)	12.52 ± 1.07 ^a^	13.92 ± 1.51 ^b^	15.04 ± 1.28 ^c^	17.03 ± 0.31 ^d^
Resistant starch (RS)	1.09 ± 0.32 ^a^	1.47 ± 0.44 ^b^	1.77 ± 0.15 ^b^	3.59 ± 1.15 ^c^

^1^ The percentages of AE addition were 0%, 25%, 50%, and 100% of the total starch. Data are presented as mean values ± standard deviation (*n* = 3). Means in the same raw are identified with superscripts (Duncan post hoc, *p* < 0.05).

**Table 2:** Semiquantitative values for sesquiterpene lactones and for the different subclasses of phenolic compounds (expressed as mg/100g) from UHPLC-QTOF-MS considering the raw (undigested) material, fermented samples and Caco-2 permeated fraction. Bioaccessibility and bioavailability are provided as % values

*mg Equivalents 100 g^−1^*				% Bioaccessibility	% Bioavailability
	Raw Material	Fermented (20 h)	Caco-2 Permeated		
Cyanidin equivalents	43.66 ± 4.74	6.24 ± 0.40	3.09 ± 0.09	14.2	50.0
Luteolin equivalents	285.82 ± 26.64	37.24 ± 0.85	14.28 ± 0.34	13.0	38.4
Catechin equivalents	102.12 ± 17.80	7.39 ± 1.18	5.28 ± 1.54	7.2	71.6
Quercetin equivalents	148.36 ± 13.61	10.71 ± 0.37	2.49 ± 0.10	7.2	23.4
Sesamin equivalents	288.30 ± 21.50	22.44 ± 0.95	12.34 ± 0.24	7.7	54.9
Chlorogenic acid equivalents	1376.90 ± 36.79	22.44 ±0.95	15.19 ± 0.53	1.6	67.6
Tyrosol equivalents	587.83 ± 99.39	24.60 ± 2.13	11.83 ± 0.45	4.2	47.9
Resveratrol equivalents	47.17 ± 2.05	3.53 ± 0.05	0.05 ± 0.00	7.4	1.4
Cynaropicrin equivalents	1199.87 ± 14.05	19.94 ± 0.65	16.36 ± 0.43	1.6	82.4

## Supplementary Materials

The following are available online at https://www.mdpi.com/2076-3921/9/4/306/s1, Table S1: dataset containing each compound identified by UHPLC-QTOF together with volcano plot on the bioavailability study.

## References

[1] Gostin A.-I., Waisundara V.Y. (2019). Edible flowers as functional food: A review on artichoke (*Cynara cardunculus* L.). Trends Food Sci. Technol..

[2] Rouphael Y., Bernardi J., Cardarelli M., Bernardo L., Kane D., Colla G., Lucini L. (2016). Phenolic compounds and sesquiterpene lactones profile in leaves of nineteen artichoke cultivars. J. Agric. Food Chem..

[3] De Falco B., Incerti G., Amato M., Lanzotti V. (2015). Artichoke: Botanical, agronomical, phytochemical, and pharmacological overview. Phytochem. Rev..

[4] Elsebai M.F., Mocan A., Atanasov A.G. (2016). Cynaropicrin: A comprehensive research review and therapeutic potential as an anti-hepatitis C virus agent. Front. Pharmacol..

[5] Lombardo S., Pandino G., Mauro R., Mauromical G. (2009). Variation of phenolic content in globe artichoke in relation to biological, technical and environmental factors. Ital. J. Agron..

[6] Colla G., Rouphael Y., Cardarelli M., Svecova E., Rea E., Lucini L. (2013). Effects of saline stress on mineral composition, phenolic acids and flavonoids in leaves of artichoke and cardoon genotypes grown in floating system. J. Sci. Food Agric..

[7] Gonthier M.P., Verny M.A., Besson C., Rémésy C., Scalbert A. (2003). Chlorogenic acid bioavailability largely depends on its metabolism by the gut microflora in rats. J. Nutr..

[8] Rocchetti G., Lucini L., Chiodelli G., Giuberti G., Gallo A., Masoero F., Trevisan M. (2017). Phenolic profile and fermentation patterns of different commercial gluten-free pasta during in vitro large intestine fermentation. Food Res. Int..

[9] Williamson G. (2017). The role of polyphenols in modern nutrition. Nutr. Bull..

[10] Manach C., Williamson G., Morand C., Scalbert A., Rémésy C. (2005). Bioavailability and bioefficacy of polyphenols in humans. I. Review of 97 bioavailability studies. Am. J. Clin. Nutr..

[11] Mahboubi M. (2018). *Cynara scolymus* (artichoke) and its efficacy in management of obesity. Bull. Fac. Pharm. Cairo Univ..

[12] Wittemer S.M., Ploch M., Windeck T., Müller S.C., Drewelow B., Derendorf H., Veit M. (2005). Bioavailability and pharmacokinetics of caffeoylquinic acids and flavonoids after oral administration of Artichoke leaf extracts in humans. Phytomedicine.

[13] Sing R.S., Singh T., Larroche C. (2019). Biotechnological applications of inulin-rich feedstocks. Bioresour. Technol..

[14] Jakobek K., Matić P. (2019). Non-covalent dietary fiber—Polyphenol interactions and their influence on polyphenol bioaccessibility. Trends Food Sci. Technol..

[15] Turkiewicz I.P., Wojdylo A., Tkacz K., Nowicka P., Hernández F. (2019). Antidiabetic, anticholinesterase and antioxidant activity vs. terpenoids and phenolic compounds in selected new cultivars and hybrids of artichoke *Cynara scolymus* L.. Molecules.

[16] Ma G., Chen Y. (2020). Polyphenol supplementation benefits human health via gut microbiota: A systematic review via meta-analysis. J. Funct. Foods.

[17] Scalbert A., Morand C., Manach C., Rémésy C. (2002). Absorption and metabolism of polyphenols in the gut and impact on health. Biomed. Pharmacother..

[18] Azzini E., Bugianesi R., Romano F., Di Venere D., Miccadei S., Durazzo A., Foddai M.S., Catasta V., Liansalata V., Maiani G. (2007). Absorption and metabolism of bioactive molecules after oral consumption of cooked edible heads of *Cynara scolymus* L. (cultivar Violetto di Provenza) in human subjects: A pilot study. Br. J. Nutr..

[19] D’Antuono I., Garbetta A., Linsalata V., Minervini F., Cardinali A. (2015). Polyphenols from artichoke heads (*Cynara cardunculus* (L.) subsp. scolymus Hayek): In vitro bio-accessibility, intestinal uptake and bioavailability. Food Funct..

[20] Englyst H.N., Veenstra J., Hudson G.J. (1996). Measurement of rapidly available glucose (RAG) in plant foods: A potential in vitro predictor of the glycaemic response. Br. J. Nutr..

[21] Camelo-Méndez G.A., Flores-Silva P.C., Agama-Acevedo E., Tovar J., Bello-Pérez L.A. (2018). Incorporation of whole blue maize flour increases antioxidant capacity and reduces in vitro starch digestibility of gluten-free pasta. Starch/Staerke.

[22] Minekus M., Alminger M., Alvito P., Ballance S., Bohn T.O., Bourlieu C., Carriere F., Boutrou R., Corredig M., Dupont D. (2014). A standardised static in vitro digestion method suitable for food—An international consensus. Food Funct..

[23] Rocchetti G., Senizza A., Gallo A., Lucini L., Giuberti G., Patrone V. (2019). In vitro large intestine fermentation of gluten-free rice cookies containing alfalfa seed (*Medicago sativa* L.) flour: A combined metagenomic/metabolomic approach. Food Res. Int..

[24] Williams B.A., Bosch M.W., Boer H., Verstegen M.W.A., Tamminga S. (2005). An in vitro batch culture method to assess potential fermentability of feed ingredients for monogastric diets. Anim. Feed Sci. Technol..

[25] Pérez-Burillo S., Rufián-Henares J.A., Pastoriza S. (2018). Towards an improved global antioxidant response method (GAR+): Physiological-resembling in vitro digestion-fermentation method. Food Chem..

[26] Hidalgo I.J., Raub T.J., Borchardt R.T. (1989). Characterization of the human colon carcinoma cell line (Caco-2) as a model system for intestinal epithelial permeability. Gastroenterology.

[27] Rocchetti G., Giuberti G., Gallo A., Bernardi J., Marocco A., Lucini L. (2018). Effect of dietary polyphenols on the in vitro starch digestibility of pigmented maize varieties under cooking conditions. Food Res. Int..

[28] Salek R.M., Steinbeck C., Viant M.R., Goodacre R., Dunn W.B. (2013). The role of reporting standards for metabolite annotation and identification in metabolomic studies. GigaScience.

[29] Garbetta A., Capotorto I., Cardinali A., D’Antuono I., Linsalata V., Pizzi F., Minervini F. (2014). Antioxidant activity induced by main polyphenols present in edible artichoke heads: Influence of in vitro gastro-intestinal digestion. J. Funct. Foods.

[30] Pandino G., Lombardo S., Williamson G., Mauromicale G. (2012). Polyphenol profile and content in wild and cultivated *Cynara cardunculus* L.. Ital. J. Agron..

[31] Camelo-Méndez G.A., Agama-Acevedo E., Sanchez-Rivera M.M., Bello-Pérez L.A. (2016). Effect on in vitro starch digestibility of Mexican blue maize anthocyanins. Food Chem..

[32] Wang S., Sun Y., Wang J., Wang S., Copeland L. (2016). Molecular disassembly of rice and lotus starches during thermal processing and its effect on starch digestibility. Food Funct..

[33] Guo P., Yu J., Copeland L., Wang S., Wang S. (2018). Mechanisms of starch gelatinization during heating of wheat flour and its effect on in vitro starch digestibility. Food Hydrocoll..

[34] Miao M., Jiang B., Jiang H., Zhang T., Li X. (2015). Interaction mechanism between green tea extract and human α-amylase for reducing starch digestion. Food Chem..

[35] Sajilata M.G., Singhal R.S., Kulkarni P.R. (2006). Resistant starch—A review. Compr. Rev. Food Sci. Food Saf..

[36] Zhu F. (2015). Interactions between starch and phenolic compound. Trends Food Sci. Technol..

[37] Camelo-Méndez G.A., Agama-Acevedo E., Tovar J., Bello-Pérez L.A. (2017). Functional study of raw and cooked blue maize flour: Starch digestibility, total phenolic content and antioxidant capacity. J. Cereal Sci..

[38] Colantuono A., Ferracane R., Vitaglione P. (2018). Potential bioaccessibility and functionality of polyphenols and cynaropicrin from breads enriched with artichoke stem. Food Chem..

[39] Sun L., Miao M. (2020). Dietary polyphenols modulate starch digestion and glycaemic level: A review. Crit. Rev. Food Sci. Nutr..

[40] Matsui T., Ogunwande I.A., Abesundara K.J., Matsumoto K. (2006). Anti-hyperglycemic potential of natural products. Mini Rev. Med. Chem..

[41] Jansen G.H.E., Arts I.C.W., Nielen M.W.F., Müller M., Hollman P.C.H., Keiker J. (2005). Uptake and metabolism of enterolactone and enterodiol by human colon epithelial cells. Arch. Biochem. Biophys..

[42] Mosele J.I., Macià A., Motilva M.-J. (2015). Metabolic and microbial modulation of the large intestine ecosystem by non-absorbed diet phenolic compounds: A review. Molecules.

[43] Thormann U., De Mieri M., Neuburger M., Verjee S., Altmann P., Hamburger M., Imanidis G. (2014). Mechanism of chemical degradation and determination of solubility by kinetic modeling of the highly unstable sesquiterpene lactone nobilin in different media. J. Pharm. Sci..

